# Modification of Cardiac Progenitor Cell-Derived Exosomes by miR-322 Provides Protection against Myocardial Infarction through Nox2-Dependent Angiogenesis

**DOI:** 10.3390/antiox8010018

**Published:** 2019-01-10

**Authors:** Seock-Won Youn, Yang Li, Young-Mee Kim, Varadarajan Sudhahar, Kareem Abdelsaid, Ha Won Kim, Yutao Liu, David J.R. Fulton, Muhammad Ashraf, Yaoliang Tang, Tohru Fukai, Masuko Ushio-Fukai

**Affiliations:** 1 Vascular Biology Center, Medical College of Georgia at Augusta University, Augusta, GA 30912, USA; swyoun93@gmail.com (S.-W.Y.); yanli@augusta.edu (Y.L.); ymkim@augusta.edu (Y.-M.K.); svaradarajan@augusta.edu (V.S.); kabdelsaid@augusta.edu (K.A.); hkim3@augusuta.edu (H.W.K.); dfulton@augusta.edu (D.J.R.F.); mashraf@augusta.edu (M.A.); yaotang@augusta.edu (Y.T.); tfukai@augusta.edu (T.F.); 2 Department of Cellular Biology & Anatomy, Augusta University, Augusta, GA 30912, USA; yutliu@augusta.edu; 3 Department of Pharmacology & Toxicology, Augusta University, Augusta, GA 30912, USA; 4 Department of Medicine (Cardiology), Augusta University, Augusta, GA 30912, USA; 5 Charlie Norwood Veterans Affairs Medical Center, Augusta, GA 30912, USA

**Keywords:** cardiac progenitor cell, myocardial infarction, exosome, miR-322, reactive oxygen species, NADPH oxidase, angiogenesis

## Abstract

Myocardial infarction (MI) is the primary cause of cardiovascular mortality, and therapeutic strategies to prevent or mitigate the consequences of MI are a high priority. Cardiac progenitor cells (CPCs) have been used to treat cardiac injury post-MI, and despite poor engraftment, they have been shown to inhibit apoptosis and to promote angiogenesis through poorly understood paracrine effects. We previously reported that the direct injection of exosomes derived from CPCs (CPCexo) into mouse hearts provides protection against apoptosis in a model of acute ischemia/reperfusion injury. Moreover, we and others have reported that reactive oxygen species (ROS) derived from NADPH oxidase (NOX) can enhance angiogenesis in endothelial cells (ECs). Here we examined whether bioengineered CPCexo transfected with a pro-angiogenic miR-322 (CPCexo-322) can improve therapeutic efficacy in a mouse model of MI as compared to CPCexo. Systemic administration of CPCexo-322 in mice after ischemic injury provided greater protection post-MI than control CPCexo, in part, through enhanced angiogenesis in the border zones of infarcted hearts. Mechanistically, the treatment of cultured human ECs with CPCexo-322 resulted in a greater angiogenic response, as determined by increased EC migration and capillary tube formation via increased Nox2-derived ROS. Our study reveals that the engineering of CPCexo via microRNA (miR) programing can enhance angiogenesis, and this may be an effective therapeutic strategy for the treatment of ischemic cardiovascular diseases.

## 1. Introduction

The transplantation of adult stem cells, including mesenchymal stem cells and cardiac progenitor cells (CPCs) has been the subject of intense investigation as a promising therapeutic strategy for cardiovascular disease (CVDs) [1,2]. While promising, a number of limitations have slowed progress. Transplanted stem cells have been shown to have limited engraftment and persistence in the harsh ischemic microenvironment post infarction [3,4]. Therefore, the functional benefits of injected stem cells have instead been proposed to arise from the release of poorly defined paracrine molecules. Increasing evidence suggests that exosomes secreted from cells play an important role in cell–cell communications via their ability to deliver biologically active cargo to adjacent/distant target cells. Exosomes are nano-size bilayer membrane vesicles (around 30~150 nm in diameter) [5] containing functional proteins, mRNA, and microRNAs (miRs). Exosomes have been shown to target ischemic myocardium, and they are capable of pronounced beneficial effects that limit heart damage and promote repair [6]. They are promising vehicles for drug, virus, gene, or miRs delivery to target cells, and they exhibit a high level of stability and efficiency [7,8]. An additional benefit of stem cell-derived exosomes is that they offer advantages in safety without compromising efficacy [9,10]. Therefore, the administration of stem cell-derived exosomes are an attractive alternative to stem cell-based therapy for the treatment of CVDs.

The miRs are a class of evolutionarily conserved, short ~22 nucleotides, non-protein-coding RNA transcripts that control gene expression via post-transcriptional repression [11]. miRs are found in exosomes, and the profile of miRs types is dependent on the cell type of origin [12]. We have recently reported that direct injection of cardiac progenitor cell-derived exosomes (CPCexo) into the ischemic heart protects against acute ischemia-reperfusion injury and this is mediated by a mechanism that enables the transfer of endogenous anti-apoptotic miRs [6,13]. The modification of stem cell-derived exosomes, or the artificial synthesis of engineered exosomes are powerful new tools that may be advantageous in treating CVDs. miR-322 has been shown to have beneficial effects in stimulating angiogenesis in tissues damaged by ischemia, such as in the post-myocardial infarction (MI) heart and the ischemic hindlimb [14]. An important target of miR-322 is CULLIN2 (CUL2), which negatively regulates the expression of hypoxia induced factor-1α (HIF1α), and a reduced expression of CUL2 increases HIF1α expression, to promote angiogenesis [14,15]. Moreover, increasing miR-322 levels in conditions of hypoxia can promote smooth muscle cell proliferation as well as cardiomyocyte protection [15,16,17]. Exosomes not only help to guide and deliver miR into target cells, but they extend the biological half-life of miR by providing protection from RNase-mediated degradation [18,19]. The exosome content can be modified by transfecting miRs into host cells, resulting in an engineered delivery system for improved efficacy [8,20]. Based on these characteristics, we hypothesized that CPCexo that were modified by enhanced expression of the pro-angiogenic miR-322 might further enhance angiogenesis and the therapeutic efficacy of CPCexo.

Reactive oxygen species (ROS) at optimal level play important physiological roles, and they are important for promoting angiogenesis in endothelial cells (ECs) [21]. Growth factors and ischemia/hypoxia increase ROS production in ECs, which promote angiogenic behaviors that include increased proliferation, migration, and capillary-like tube formation [21,22,23]. The NADPH oxidase (NOX) family is one of the major sources of ROS in the vasculature. In ECs, NOX-derived ROS are derived from either Nox2 or Nox4, and both have been shown to be important for endothelial angiogenesis [24,25,26,27,28,29]. However, a role of ROS in the beneficial effects of exosomes in driving angiogenic responses in ECs has never been reported.

In this study, we show that systemic administration of bioengineered CPCexo enriched with the pro-angiogenic miR-322 elicits enhanced angiogenesis and increased protection against MI-induced cardiac injury, as compared to control CPCexo. The mechanism by which CPCexo-322 promotes angiogenesis is mainly via the enhancement of the Nox2-ROS signaling axis. Our study supports the concept that genetic programming of stem cell derived exosomes to include miRs endowed with unique functions is a promising therapeutic strategy for the treatment of CVD.

## 2. Materials and Methods

All experimental procedures were performed in accordance with the National Institute of Health (NIH) Guide for the Care and Use of Laboratory Animals, and approved by the Institutional Animal Care and Use Committee of Augusta University (protocols 2013-0537).

### 2.1. Mouse CPCs Isolation

CPCs were isolated from the hearts of 2-month-old male, C57BL/6 mice (The Jackson Laboratory, Bar Harbor, ME, USA) via a 2-step procedure that has been described previously [6,30,31]. Briefly, in step 1, cardiac explants were minced under sterile conditions and cultured for 2–3 weeks until small, round, phase-bright cells had migrated from the adherent explants and proliferated over a fibroblast layer. In step 2, Sca-1 + cells were isolated from the phase-bright cells using a magnetic-activated cell sorting (MACS) approach with Sca-1 magnetic beads (Miltenyi Biotec Inc., Auburn, CA, USA) as per the manufacturers’ protocols. The selected Sca-1 cells were cultured and maintained in complete media containing Dulbecco’s Modified Eagle Media (DMEM)/F12, 10% exosome-free fetal bovine serum (Exo-FBS, System Biosciences (SBI), Mountain View, CA, USA), 200 mM l-glutamine, 55 nM β-mercaptoethanol, and 1% MEM nonessential amino acids.

### 2.2. Exosome Collection

The isolation of CPC-exosomes was performed at 4 °C as described elsewhere, with slight modification [6]. In brief, isolated CPCs were cultured in 20 mL culture medium (CM) with 10% exosome-depleted FBS. After 48 h, the supernatant was centrifuged at 300× *g* for 10 min to eliminate cells, followed by filtration through a 0.22 µm low protein binding filter (Millipore, Temecula, CA, USA) to remove cell debris, as well as microvesicles. Exosomes were then isolated using ultracentrifugation at 100,000× *g* for 90 min, and then resuspended in 100 µL of phosphate-buffered saline (PBS) per tube, and stored at −80 °C.

### 2.3. Immuno-Transmission Electron Microscopy

Standard immunohistochemical staining was performed with anti-CD63 antibodies, as described previously [32]. Fixed exosome pellets were incubated on carbon–formvar-coated 200 mesh nickel grids for 30 min. Grids were subjected to a 30 min quench with 1 M ammonium chloride, and then blocked with 0.4% Bovine serum albumin (BSA, Sigma-Aldrich, St. Louis, MO, USA) in PBS for two hrs. Grids were then incubated with primary rabbit-anti-CD63 antibody (1:100, SC-15363, Santa Cruz Biotechnology, Santa Cruz, CA, USA) for 1 hr, and then floated on drops of 1.4 nm anti-rabbit nanogold (1:1000, Nanoprobes, Inc., Yaphank, NY, USA) in blocking buffer for 1 hour. After enhancement with HQ Silver (gold enhancement reagent, Nanoprobes, Inc., Yaphank, NY, USA), samples were allowed to air-dry, and observed under a transmission electron microscope (JEOL JEM 1230, Peabody, MA, USA).

### 2.4. Nanoparticle Tracking Analysis (NTA) with ZetaView

Isolated exosomes were diluted using 1 × PBS buffer, and particle sizes and concentrations were measured as described previously [13]. NTA measurements were recorded and analyzed at 11 positions. The ZetaView system was calibrated using 100 nm polystyrene particles. The temperature was maintained between 23–37 °C. Zeta potential was measured using 0.05 × PBS instead of 1 × PBS to adjust conductivity to around 500 μS/cm.

### 2.5. Exosome Modification by Transfecting MicroRNAs Using Electroporation

To modify exosomes with miR-Con or miR-322 (Exiqon, Germantown, MD, USA), CPCexo was incubated with miR-322 (10 pmole/µg) in PBS, which resulted in 10 nM of miR-322 in CPCexo, because exosome density was around 1.15–1.19 g/mL [33]. Using the Neon transfection system (Invitrogen, Carlsbad, CA, USA), miR-322 was incorporated into CPCexo using settings of 5 mV for 20 s. After electroporation, the exosomes were diluted with 1× PBS and ultracentrifuged at 100,000× *g* for 75 min. The exosomes were then resuspended with 1 × PBS. The transfection efficiency of miRs was evaluated by relative expression of miR-322 levels in CPCexo with and without miR-322 transfection, using quantitative polymerase chain reaction (qPCR). We injected 10 µg of CPCexo-322/mice, corresponding to 5.9~7.1 µg/mL for in the vivo experiments, and we used CPCexo-322 (5.0 µg/mL) for in the vitro experiments.

### 2.6. Human Umbilical Vein Endothelial Cell Culture Treated with Exosomes, or Transfected with miRs or siRNAs

Human umbilical vein endothelial cells (HUVECs, Lonza, Basel, Switzerland) were used between passage 4 to 6 and cultured with EndoGRO (Millipore, Temecula, CA, USA) including 5% fetal bovine serum (FBS, Atlanta Biologicals, Flowery Branch, GA, USA) and supplements. HUVEC were incubated with isolated exosomes for 24 h, and cell media were changed to EndoGRO with 0.5% FBS for overnight. To transfect miRs and small interfering RNA (siRNA) into HUVECs, 30 nM miRs or 20 nM siRNA were incubated with Lipofectamine RNAi Max and Oligofectamine (Invitrogen, Carlsbad, CA, USA), respectively in OPTI-MEM (Invitrogen, Carlsbad, CA, USA), as per the manufacturer’s instructions. 

### 2.7. Measurement of EC Migration (Modified Boyden Chamber Assay)

To analyze cell migration, we used a modified Boyden chamber assay, as previously reported [34,35,36]. Serum-starved HUVECs (6 × 10^4^ cells) were seeded on 0.1% gelatin-coated transwell inserts with pores sizes of 8 µm (Falcon, Tewksbury, MA, USA), and transferred to the lower chamber in a 24-well plate that contained EndoGRO medium with 0.5% FBS. The plates were incubated for 6 h in 37 °C in a 5% CO_2_ incubator. Migrating cells were fixed with 4% paraformaldehyde (PFA) and stained with 10% crystal violet (Sigma-Aldrich, St. Louis, MO, USA) after removing the non-migrating cells present on the top of the membrane with wet cotton swabs. Cells that had migrated through the membranes in the bottom of transwell inserts were then counted.

### 2.8. Measurement of Capillary Tube Formation on Matrigel

To measure capillary tube formation on Matrigel, serum-starved HUVECs (3 × 10^4^ cells) were used as we have previously reported [37,38]. Growth factor-reduced Matrigel (BD Biosciences, San Jose, CA, USA) was used to coat 48-well plates, and cells were seeded on the solidified Matrigel. After 6 h, the number of tubes was analyzed using ImageJ.

### 2.9. Uptake of PKH67-Labeled Exosomes by HUVECs

Purified CPC-exosomes were labeled using the PKH67 green fluorescent labeling kit (Sigma-Aldrich, St. Louis, MO, USA) according to the manufacturer’s instructions. The PKH67 concentration used for exosome labeling was 2 μM. The labeled exosomes were stained with PKH67 dye in 400 µL Diluent C fluid supplied with the kit for 5 min at room temperature, and an equal volume of exosome free serum was added to stop the labeling. The exosomes were re-purified via ultracentrifugation (100,000× *g*, 75 min). The labeled CPC-exosomes were incubated on HUVECs for 2 h at 37 °C, and cells were washed using PBS. The uptake of exosomes in HUVECs was evaluated using a fluorescence microscope (Keyence, BZ-X700, Osaka, Japan). The fluorescence intensity was measured using ImageJ.

### 2.10. Measurement of Reactive Oxygen Species

HUVECs were incubated with 20 μM M-H2DCFDA (5-(and-6)-chloromethyl-2′,7′-dichlorodihydrofluorescein diacetate, acetyl ester, Invitrogen, Carlsbad, CA, USA) for 6 min at 37 °C, fixed with 4% PFA for 10 min at room temperature (RT), as previously reported [34,36]. The cells were mounted using VECTASHIELD mounting medium with 4′,6-diamidino-2-phenylindole (DAPI, Vectorlabs, Burlingame, CA, USA). The extent of DCF fluorescence in cells was measured by fluorescence microscopy (Keyence, BZ-X700, Osaka, Japan) and analyzed using ImageJ.

### 2.11. Myocardial Infarction Model and Exosome Injection

Mice were subjected to ligation-induced MI, as described previously [39]. Briefly, male C57BL/6 mice weighing 25–30 g were anesthetized with intraperitoneal 125 mg/kg ketamine combined with 62.5 mg/kg xylazine. Mice were intubated with a 24-gauge tube in the trachea, and ventilated using a Harvard rodent ventilator (Inspira Advanced Safety Ventilator Model 55-7058, Holliston, MA, USA). The chest was opened via a lateral thoracotomy, and the heart was exposed through a pericardiotomy. An 8-0 nylon suture (Monosof) was placed under the left anterior descending artery (LAD) and ligated. The chest was closed, and the mice were allowed to recover. Based on previous published studies [40,41], we elected to use 10 µg exosomes/mouse and mice were injected with CPC-exosomes (10 µg/50 µL) via the tail vein at 3, 7, and 11 days after surgery. Mice were then assigned to three groups after injection (N = 5 per group): a PBS, CPCexo-Con, and a CPCexo-322 group. After 14 days post-MI, the mice were sacrificed for histology.

### 2.12. Measurement of the Cardiac Infarct Area

At 14 days, mouse hearts were fixed with 4% parafarmaldehyde overnight, and then embedded in optimum cutting temperature (OCT, Fisher Health Care, Pittsburgh, PA, USA). Sections 7 µm thick were cut using a cryostat (Thermo Scientific, Waltham, MA, USA), and stained with Masson’s Trichrome according to the manufacturer’s instructions (Thermo Scientifics). To measure the cardiac infarct area and LV volume, we used ImageJ. The cardiac infarct ratio was calculated by dividing the area of infarct by the total heart area. The LV volume ratio was calculated by dividing the LV chamber size by total heart area (N = 5). To analyze capillary density, sections were stained using anti-biotinylated ILB4 antibody and anti-streptavidin-HRP (Vectorlabs, Burlingame, CA, USA). DAP (Vectorlabs, Burlingame, CA, USA) was used for the chromophore and hematoxylin (Sigma-Aldrich, St. Louis, MO, USA) was used for nucleus stain, and the number of capillaries was counted. To quantify angiogenesis in heart, the number of capillaries and vessels were analyzed in the border zone using ImageJ. 

### 2.13. Quantitative Polymerase Chain Reaction

Total RNA was isolated from exosomes and HUVECs by using the TRI Reagent (Molecular Research Center Inc., Cincinnati, OH, USA). A total of 2 µg of total RNA from HUVEC was reverse transcribed by a high capacity cDNA reverse transcription kit (Applied Biosystems, Foster City, CA, USA). The miRs transcript II kit (Qiagen, Venlo, The Netherlands) was used to make miRs cDNA from total RNA isolated from 100 µg of exosomes using. Exactly 40 ng of complementary DNA (cDNA) and 1 µL of miRs cDNA were used to measure gene and miR expression, respectively, using qPCR and SYBR Green detection (Qiagen, Venlo, The Netherlands) on the ABI Prism 7000. The 2^-ddCT method was used to calculate differences in miR expression. Nox2 primers, forward 5′-AGC TAT GAG GTG ATG TTA GTG G-3′, reverse 5′-TGC AAT ATT TGT ACC AGA CTG ACT TGA G-3′; Nox4 primers, forward 5′-CTC AGC GGA ATC AAT CAG CTG TG-3′, reverse 5′-AGA GGA ACA CGA CAA TCA GCC TTA G-3′; 18S ribosomal RNA (rRNA) primers, forward 5′-GCT TAA TTT GAC TCA ACA CGG GA-3′, reverse 5′-AGC TAT CAA TCT GTC AAT CCT GTC-3′, as we have previously reported [34]. Gene expression and miR expression were normalized by 18S rRNA (internal control for mRNA) and RNU6 snRNA (internal control for miR) [20,42,43], respectively. Data were expressed by the fold-change from the controls such as non-treatment, CPCexo-Con, or CPCexo-Con, with siControl.

### 2.14. Statistical Analysis

Results are expressed as mean ± S.E. Statistical significance was assessed by Student’s paired two-tailed *t*-test or an analysis of variance on untransformed data, followed by a comparison of the group averages by contrast analysis, using the Super ANOVA statistical program (Abacus Concepts, Berkeley, CA, USA). A *p* value of <0.05 was considered to be statistically significant, and a *p* value of <0.01 was considered to be more statistically significant.

## 3. Results

### 3.1. Characterization of CPC-Derived Exosomes

**Figure 1:**
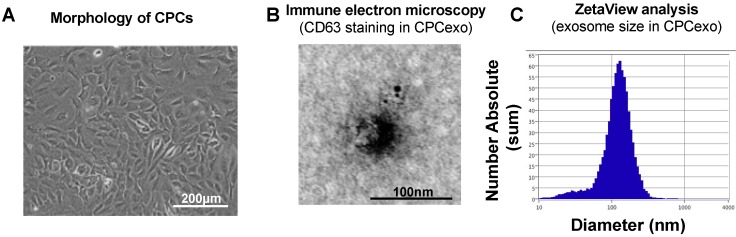
Isolation and characterization of exosomes from mouse cardiac progenitor cells (CPCs). (**A**) Morphology of cultured mouse CPCs. The scale bar is 200 µm. (**B**) Immune electron microscopy image for CD63 in isolated CPCexo. The scale bar is 100 nm. (**C**) ZetaView analysis for the size and concentration of isolated CPCexo. The y-axis represents the absolute number of exosomes at each particle size, and the x-axis represents the size of the exosomes (diameter/nm).

To investigate the function of mouse CPCexo, we isolated CPCs from C57BL/6 mouse hearts (Figure 1A) using a hematopoietic lineage-depletion cocktail, followed by enrichment for Sca-1+ cells via Magnetic-activated cell (MACS) sorting, as we have previously reported [6]. We confirmed that isolated CPCs express Sca-1, CD105, and the early cardiac transcription factor, GATA4 (data not shown). We then isolated CPCexo from the isolated CPCs by using a 0.22 μm filter system and ultracentrifugation, as described in the Methods section. The isolated exosomes (Figure 1A) were analyzed with immune-electron microscopy imaging for CD63 and ZetaView (Figure 1B,C). The isolated exosomes from CPCs (CPCexo) were positive for the exosome marker CD63, as determined by transmission electron microscopy. The average size and diameter of CPCexo were 127.8 nm and 128.0 nm respectively, indicating that the majority of particles that were recovered were within the size range of exosomes (50–150 nm).

### 3.2. Transfection of miR-322 into CPC-Derived Exosomes (CPCexo)

**Figure 2:**
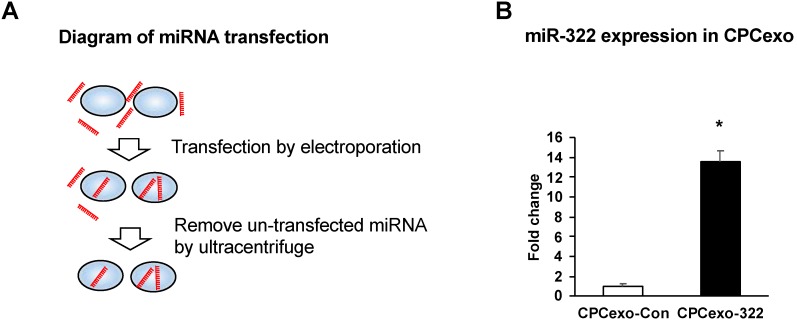
CPC-derived exosomes are transfected with miR-322. (**A**) Diagram of the miR transfection protocol using electroporation. (**B**) The relative ratio of miR-322 expression in CPCexo transfected with the control miR (CPCexo-Con) or miR-322 (CPCexo-322) relative to RNU6 snRNA, an endogenous control (*n* = 3, * *p* < 0.05 (CPCexo-Con vs. CPCexo-322)).

We next transfected miR-control (miR-Con) or miR-322 into CPCexo using electroporation (Figure 2A). We found that CPCexo transfected with miR-322 (CPCexo-322) had significantly higher levels of miR-322 expression, as compared to CPCexo transfected with the miR-Con (CPCexo-Con) (Figure 2B). These results suggest that isolated exosomes can be modified by the transfection of miRs. 

### 3.3. Beneficial Effects of CPCexo-322 in the Myocardial Infarction Model

**Figure 3:**
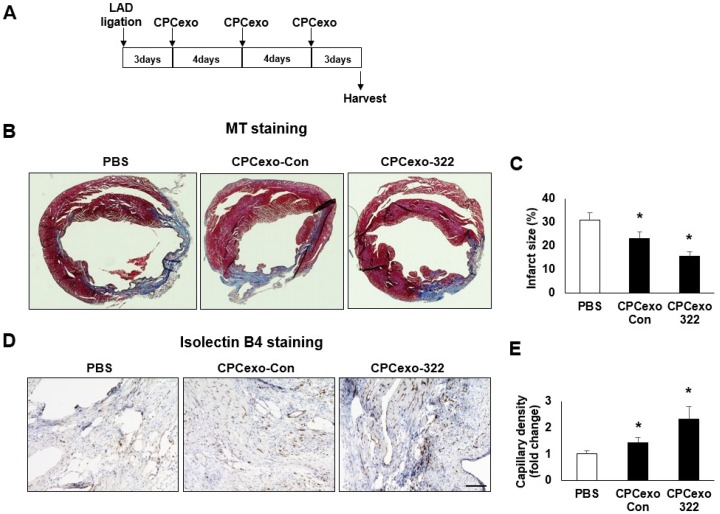
CPCexo-322 reduced the area of myocardial infarction-induced injury after LAD ligation. (**A**) Schematic outline of the experimental strategy and schedule of CPCexo III injections post LAD ligation. (**B**) Masson’s trichrome stain (MT) of MI heart cross-sections. Blue area indicates fibrosis. (**C**) Quantitative analysis of infarct size (*n* = 5). (**D**) Immunohistochemical staining with isolectin B4 (ILB4, brown) and nuclei (hematoxylin, blue). Scale bar is 100 µm. (**E**) quantitative analysis of capillary density on the border zone assessed by isolectin B4 (ILB4) staining (*n* = 5, * *p* < 0.05 (PBS vs. CPCexo), and ns; not significant).

To evaluate the effects of the CPCexo-322 on cardiac injury in the MI model, we injected CPCexo-322, CPCexo-Con (10 µg/50 µL) or PBS (control) via the tail vein at 3, 7, and 11 days after ligation of the left anterior descending (LAD) artery (Figure 3A). The mice were sacrificed at 14 days and evaluated for MI area and left ventricle dilation. The hearts in the PBS injected group had a larger infarct area compared to the CPCexo-Con injected hearts. Masson’s trichrome (MT) staining revealed an increase in collagen deposition and the organization of collagen fibrils (blue regions) (Figure 3B). The fibrotic region was greatly increased in the PBS injected group as compared to the CPCexo-Con injected group. CPCexo-322 further decreased the infarct area as compared to CPCexo-Con (Figure 3C). Angiogenesis after MI is a critical factor to rescue damaged cardiomyocytes and reduce fibrosis [44,45]. Therefore, we investigated whether CPCexo-322 enhanced angiogenesis in the infarct zone by estimating capillaries and vessels with endothelial markers (Isolectin B4) (Figure 3D). Capillary density was dramatically increased in CPCexo-322 injected group compared to CPCexo-Con and PBS groups (Figure 3E). These results suggest that CPCexo-322 enhances myocardial angiogenesis in the infarcted area.

### 3.4. Uptake of CPCexo by Cultured ECs and Enhanced Angiogenesis by CPCexo-322

**Figure 4:**
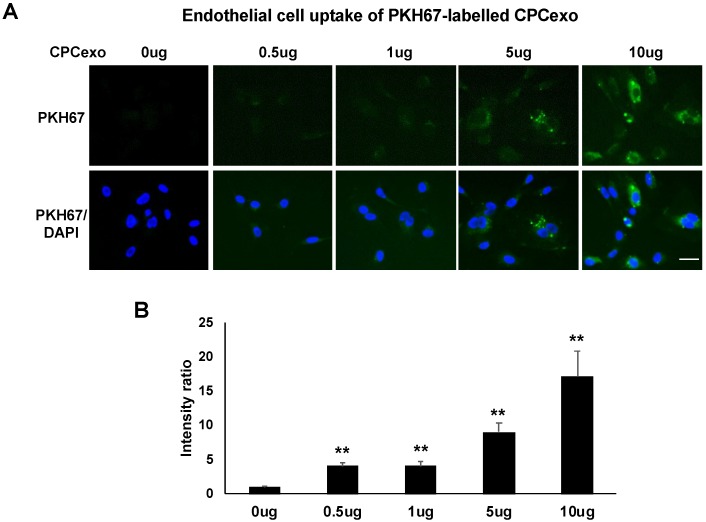
Uptake of CPCexo by human ECs. (**A**) The fluorescence images of PKH67 (Green) labeled CPCexo added to HUVECs for 2 h at the indicated concentrations. Nuclei were stained with DAPI (Blue) to show the cell confluency. (**B**) Averaged fluorescence intensity of PKH67 positive CPCexo incorporated by HUVECs in a dose-dependent manner. The scale bar is 20 μm (*n* = 3, ** *p* < 0.01 (0 µg vs. each dose)).

Next, we investigated how CPCexo-322 increases angiogenesis using cultured human umbilical vein endothelial cells (HUVECs). We first examined the transfer efficiency of CPCexo into HUVECs using CPCexo labelled with PKH67, a fluorescent cell linker compound that is incorporated into cell membranes. Figure 4A,B demonstrate that PKH67-labelled CPCexo were taken up by HUVECs in a dose-dependent manner (5–10 µg/mL). Since CPCexo at concentrations of 5.9~7.1 µg/mL were effective in in vivo experiments, we used a similar concentration (5 µg/mL) of CPCexo for in vitro angiogenesis experiments.

**Figure 5:**
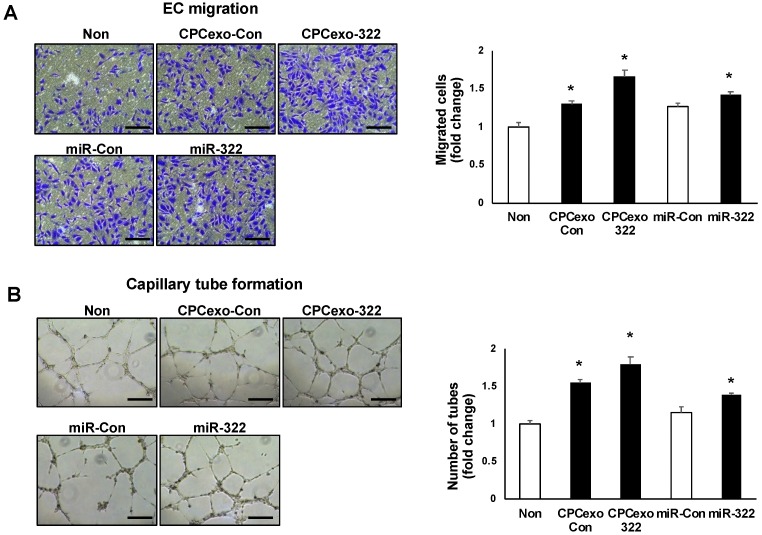
CPCexo-322 increases angiogenesis in ECs. (**A**,**B**) HUVECs were treated with or without CPCexo-Con or CPCexo-322. (**A**) A modified Boyden Chamber assay was used to measure EC migration. Bar graphs represent the average number of migrated cells per five random fields and is expressed as the fold change over baseline (non-treated group). (**B**) Endothelial cell capillary network formation assay on growth factor-reduced Matrigel. Images were using with a digital camera. Bar graphs represent the average number of tubes per five random fields per well and is expressed as the fold change over control (non-treated group). The scale bar is 50 μm (*n* = 3, * *p* < 0.05 (Non vs. CPCexo or miR-Con vs. miR-322)).

We next examined whether CPCexo-322 promotes angiogenic behaviors in endothelial cells such as cell migration using modified Boyden Chamber (Figure 5A) as well as capillary-tube formation on Matrigel (Figure 5B). CPCexo-322 treatment significantly increased both cell migration and tube formation as compared to CPCexo-Con which also showed significant increases in both responses as compared to the non-treated control group. We confirmed that miR-322 transfection to HUVECs increased both angiogenic responses versus the miR-Con-transfected cells. Both increased angiogenic responses to a lesser extent than CPCexo-322 (Figure 5A,B). These results suggest that CPCexo-322 promotes angiogenesis more effectively than CPCexo-Con or miR-322 alone in cultured human ECs.

### 3.5. CPCexo-322 Increase ROS Production via the Upregulation of Nox2 in ECs

We next investigated the mechanisms underlying the enhanced efficacy of CPCexo-322-in promoting angiogenesis. miR-322 has been shown to decrease CUL2 expression and promote angiogenesis through stabilization of HIF1α protein expression [14]. Therefore, we first examined if CPCexo-322 similarly regulates CUL2 expression using qPCR. miR-322 transfection of HUVECs resulted in reduced Cul2 mRNA and protein expression and increased HIF1α protein expression as compared to the miR-Con group (Supplementary Figure S1B,C) and as reported [14]. In contrast, CPCexo-Con or CPCexo-322 treatment increased Cul2 mRNA expression without altering HIF1α or Cul2 protein expression compared to non-treated HUVECs in normoxic condition (Supplementary Figure S1A,C). These results suggest that CPCexo-322 increases angiogenic responses in ECs through mechanisms that are independent of the miR322-CUL2-HIF1α signaling axis.

**Figure 6:**
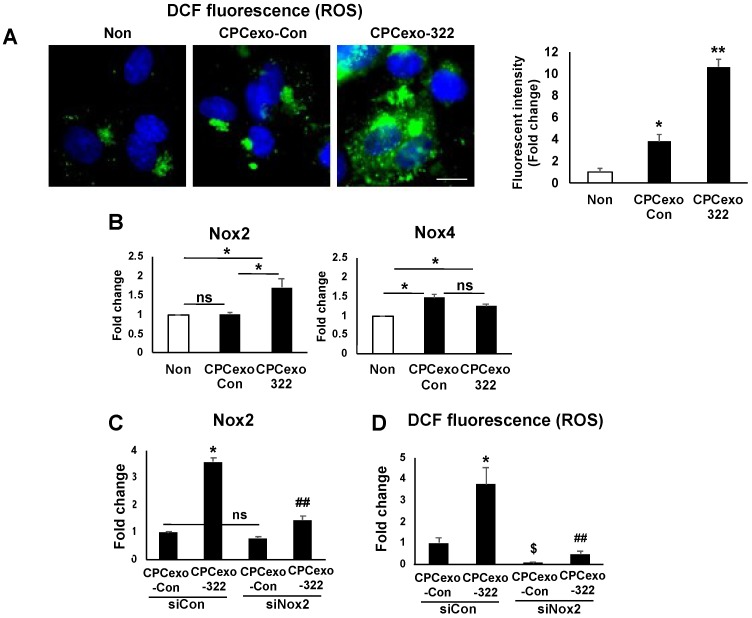
CPCexo-322 increases ROS via upregulating Nox2 in ECs. (**A**) DCF fluorescence images in HUVECs treated with CPCexo-322 or CPCexo-Con or no treatment (Non). Nucleus was stained with DAPI (Blue). The scale bar is 10 µm. The fluorescence intensity was measured using Image J. (**B**) Nox2 or Nox4 mRNA expression in HUVECs treated with CPCexo-322 or CPCexo-Con or no treatment (Non). (**C**,**D**) Nox2 mRNA expression (C) or DCF fluorescence (D) in siControl- or siNox2-transfected HUVECs treated with CPCexo-Con or CPCexo-322. Graphs represent the fold change from control (non-treated groups for A and B; siControl + CPCexo-Con groups for C and D) (*n* = 3, * *p* < 0.05 (Non vs. CPCexo or CPCexo-Con vs. CPCexo-322), ## *p* < 0.01 (siCon vs. siNox2 in CPCexo-322); $ *p* < 0.05 (siCon vs. siNox2 in CPCexo-Con); and ns (not significant)).

Since we and others reported that ROS, and especially H_2_O_2_, derived from NADPH oxidase (NOX) promote angiogenesis in ECs [34,36,46,47,48], we next examined if CPCexo-322 alters ROS levels in ECs. CPCexo-322 (Figure 6A) or miR-322 (Supplementary Figure S2A) treatment in HUVECs significantly increased DCF fluorescence a probe that reflects intracellular redox status. DCF signal was abolished by a cell-permeable PEG-catalase (Supplementary Figure S3) indicating its specificity for H_2_O_2_. Of note, CPCexo-Con also increased DCF fluorescence but these changes were much less pronounced than CPCexo-322 (Figure 6A and Supplementary Figure S3). These results suggest that CPCexo-322 or miR-322 can increase intracellular H_2_O_2_ levels to a greater extent than CPCexo-Con in ECs.

Given that ROS derived from Nox2 and Nox4 are known to play an important role in angiogenesis in ECs [24,25,26,27,28,29], we next examined whether CPCexo-322 or CPCexo-Con regulate Nox2 or Nox4 expression in HUVECs. We found that CPCexo-322, but not CPCexo-Con, significantly increased Nox2 mRNA while CPCexo-Con or CPCexo-322 slightly increased Nox4 mRNA to a similar extent compared to control (Figure 6B). Of note, miR-322 alone significantly increased Nox2 mRNA without altering Nox4 mRNA levels (Supplementary Figure S2B). Furthermore, Nox2 siRNA significantly inhibited CPCexo-322-induced increases in Nox2 mRNA (75%, Figure 6C) and DCF fluorescence (80%, Figure 6D). These results suggest that CPCexo-322 treatment in ECs increases ROS production via miR-322-mediated upregulation of Nox2.

### 3.6. CPCexo-322 Induces Angiogenic Responses in ECs via the Upregulation of Nox2

**Figure 7:**
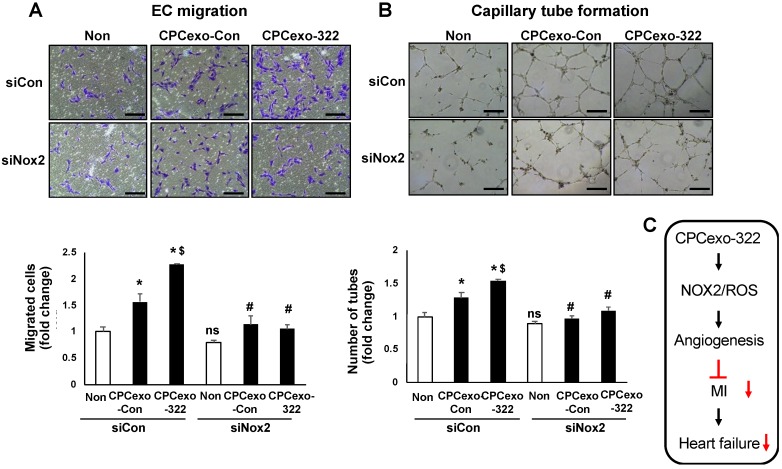
Nox2 is critical for CPCexo-322-induced angiogenic responses in ECs. (**A**,**B**) HUVECs were transfected with siControl (siCon) or siNox2 and then treated with or without CPCexo-Con or CPCexo-322. In A, cells were cultured in a modified Boyden Chamber to measure EC migration. Bar graphs show the average number of cells migrated per five random fields and is expressed as the fold change over the control (siCon with non-treated group). In B, the ability of endothelial cells to form capillary networks was determined using a Matrigel assay. Images were taken with a digital camera. Bar graphs represent the average number of tubes per five random fields per well and data is expressed as the fold change over control (siCon with non-treated group). The scale bar is 50 μm (*n* = 3, * *p* < 0.05 (Non vs. CPCexo-con/322); # *p* < 0.05 (siCon vs. siNox2 in each CPCexo); $ *p* < 0.05 (CPCexo-Con vs. CPCexo-322); and ns; not significant (siCon vs. siNox2 in Non)).

Our next goal was to examine the functional significance of CPCexo-322-induced upregulation of Nox2 in mediating the enhanced angiogenesis seen in ECs. Figure 7 shows that both CPCexo-322- and CPCexo-Con-induced increases in EC migration (Figure 7A) and capillary tube formation on Matrigel (Figure 7B) were almost completely inhibited by Nox2 siRNA.

## 4. Discussion

CPCs have been proposed as a promising therapy to limit cardiac damage post MI and promote repair. However, in the absence of stable engraftment of CPCs, questions have arisen about how exactly they improve function and the narrative has shifted towards a role for paracrine factors. The cardiac origin of CPCs is thought to provide unique advantages with the release of a unique array of paracrine molecules that are effective in preventing apoptosis in cardiac myocytes and stimulate new blood vessel formation. Exosomes have been shown to mediate cell-cell communication and transport signaling molecules in the form of proteins and RNA. We have previously reported that the direct injection of CPCexo into ischemic hearts provides protection against apoptosis by transferring endogenous anti-apoptotic miRs [6,13]. In this study, we bioengineered CPCexo with modified pro-angiogenic miR-322, with the goal of improving efficacy, and assessed the functional differences in a mouse MI model following systemic injection of CPCexo. We found that injection of CPCexo-322 significantly reduced infarct area, left ventricular dilation, fibrosis and increased angiogenesis at ischemic border zones more efficiently than CPCexo-Con. Mechanistically, CPCexo-322 increased ROS production in cultured human endothelial cells, increased migration, and capillary network formation and these effects were mediated by enhanced NOX2 expression. Collectively, these findings suggest that miR-322 modified CPCexo provide additional beneficial effects beyond those of CPCexo-Con, by selectively enhancing Nox2-ROS-dependent therapeutic angiogenesis which protects against MI-induced cardiac injury.

Analysis of exosome composition using immune-electron microscopy and Zetaview analysis revealed that the exosomes that were purified from the conditioned medium of CPCs expressed the exosome marker, CD63, and they have the expected diameter, confirming the successful isolation of exosomes from CPCs. They also displayed behaviors that were consistent with endosomes, as PKH67-labelled CPCexo were efficiently taken up into HUVECs. Because exosomes have a bilayer membrane that is similar to the cell membrane [49,50], we used electroporation to transfect miRs into isolated exosomes. Using this method, we transfected CPCexo with miR-322, which is the rodent homolog of human miR-424, increasing the expression levels by 14-fold. These results suggest that miR levels in exosomes can be modified after isolation, which may benefit approaches in vivo to delivering therapeutic exosomes in both animal models and clinical approaches.

In our prior study, we found that CPCexo contain high level of anti-apoptotic miR-451 and that direct injection of these exosomes in ischemic hearts provided protection against apoptosis. Our current study provides novel evidence that the therapeutic effects of CPCexo can be enhanced by transfection with miR-322, which has been shown by others to promote angiogenesis by blocking the degradation of HIF1α [14]. We had previously shown that the IV delivery of exosomes after MI resulted in their accumulation in the ischemic zone after 1 hour [41] and additional CPCexo injections at later times provided additional protection against cardiac injury [51]. To maximize cardiac benefits, we pursued a similar strategy of injecting CPCexo-322 at multiple time points, post-MI. We found that CPCexo-322 significantly reduced the size of the infarcted area, and increased angiogenesis in the ischemic border zone more efficiently than CPCexo-Con. These results suggest that the systemic delivery of miR-modified CPC-derived exosomes is an improved therapeutic approach for countering ischemic damage in cardiac diseases such as MI.

To determine the mechanisms by which CPCexo-322 enhances angiogenesis, we first investigated the established targets of miR-322. miR-322 has been reported to target CUL2, and increased miR-322 levels promote angiogenesis by reducing CUL2 expression and increasing HIF1α stability [14]. We found that miR-322 transfection did indeed reduce CUL2 expression in ECs, but we also found that CPCexo-322 or CPCexo-Con treatment unexpectedly increased CUL2 expression in HUVECs. These results suggest that CPCexo-322 promotes angiogenesis through a mechanism that is independent of the miR322-CUL2-HIFα axis in ECs. The underlying reasons for these differences are not known but could relate to the delivery system, as exosome encapsulated miRs are likely more stable, and they are protected against RNase-mediated degradation [8], or that the CPCexo contains a multitude of regulatory RNAs and other factors that alter the targeting profile [7].

We and others have reported that NOX-derived H_2_O_2_ is required for angiogenic responses in ECs and post-ischemic neovascularization in vivo [24,25,26,27,28,29]. We next investigated whether changes in ROS explains the enhanced efficacy of CPCexo-322 from CPCexo-Con. In the present study, we found that CPCexo-322 increased angiogenesis through Nox2-dependent H_2_O_2_ production. In EC exposed to CPCexo-Con, Nox4 expression was slightly upregulated without affecting Nox2 expression. In line with these findings, previous studies have shown that Nox2-derived ROS are required for myocardial angiogenesis and cardioprotection [52]. We found that the ability of CPCexo-322 to induce EC migration and tube formation were inhibited by Nox2 siRNA. We have previously validated the ability of this siRNA to knockdown Nox2 expression without altering Nox4 expression in HUVECs [34]. Given that Nox4-derived H_2_O_2_ has been shown to be an upstream regulator of Nox2 activation in ECs [34], these results suggest that the ability of CPCexo-322- and CPCexo-Con to induce angiogenic responses are mediated through Nox2-dependent mechanisms. CPCexo-322, but not CPCexo-Con, increases Nox2 expression which may explain its greater efficacy.

The mechanisms whereby CPCexo-322 and miR-322, but not CPCexo-Con, increases Nox2 without altering Nox4 expression, remain unclear. Our computational target prediction analysis indicates that mmu-miR-322 or its human homolog, hsa-miR-424 does not directly bind to the 3′-untranslated regions of any genes associated with Nox2 or its regulation. Thus, the ability of both CPCexo-322 and miR-322 to induce the upregulation of Nox2 in HUVECs must be mediated through a yet to be defined indirect mechanism. Future studies will be necessary to identify the direct downstream targets of CPCexo-322 that are responsible for increasing Nox2 transcription.

In summary, we have demonstrated that isolated exosomes from CPCs can be modified by electroporation-driven uptake of pro-angiogenic miRs, yielding modified exosomes that have improved beneficial effects on cardiac injury through the stimulation of angiogenesis, and the attenuation of infarct size in a mouse model of MI. We have also unveiled a novel mechanism by which CPCexo-322 promotes angiogenesis, the upregulation of the Nox2-ROS signaling axis. Thus, bioengineering CPCexo with pro-angiogenic miRs may be an attractive therapeutic approach to promote vascularization and regeneration in ischemic diseases.

## Supplementary Materials

The following are available online at http://www.mdpi.com/2076-3921/8/1/18/s1, Figure S1: The effects of CPCexo-322 and miR-322 on CULLIN2 (CUL2) expression, etc.
