# Supplementary material for: Modification of Cardiac Progenitor Cell-Derived Exosomes by miR-322 Provides Protection against Myocardial Infarction through Nox2-Dependent Angiogenesis
Source: Antioxidants (Basel). 2019 Jan 10;8(1):18. doi: 10.3390/antiox8010018 (PMC6356993; doi:10.3390/antiox8010018)
Supplement: Supplementary file 1 [file antioxidants-08-00018-s001.pdf]

Supplementary Material:

Materials and Methods

Quantitative Polymerase Chain Reaction

Total RNA from HUVECs was isolated with TRI Reagent (Molecular Research Center Inc.) per the manufacturer's instructions. 2 μg of total RNA was used to make cDNA using a high capacity cDNA reverse transcription kit (Applied biosystems). 40ng of cDNA was used to analyze gene expression using the SYBR Green PCR kit (Qiagen) and the ABI Prism 7000. Cullin 2 (Cul2) primers, forward 5′-CTT ACT CCG TGC TGT GTC CA-3′, reverse 5′-GCC TTA TCC AAC GCA CTC AT-3′. Gene expression was normalized and is expressed as fold change relative to 18S rRNA (internal control).

Immunoblot

HUVEC total protein was isolated in RIPA buffer [50 mM HEPES (pH 7.4), 5 mM EDTA, 120 mM NaCl, 1% Triton X-100, protease inhibitors (10 g/ml aprotinin, 1 mM phenylmethylsulfonyl fluoride, 10 g/ml leupeptin) and phosphatase inhibitors (50 mM sodium fluoride, 1 mM sodium orthovanadate, 10 mM sodium pyrophosphate)]. To evaluate HIF1α expression, cells were treated with 100 μM of CoCl2 (Sigma). Cell lysates were used for immunoblotting with anti-HIF1α (BD Bioscience), anti-Cul 2 (Santacruz) and GAPDH (GeneTex).

Measurement of Reactive Oxygen Species (ROS)

HUVECs were incubated with CPCexo-Con and CPCexo-322 overnight, with and without polyethylene glycol (PEG)-catalase (100 U/ml) for 24 hours. Cells were then incubated with 20 μM CM-H2DCFDA (5-(and-6)-chloromethyl-2′,7′-dichlorodihydrofluorescein diacetate, acetyl ester, Invitrogen) for 6 min at 37 °C, fixed with 4% PFA for 10 min at room temperature. Treated cells were mounted using the VECTASHIELD mounting medium together with DAPI. DCF fluorescence was measured by fluorescence microscopy. The degree of DCF fluorescence in DAPI positive cells was analyzed using Image J (NIH, Bethesda, MD).

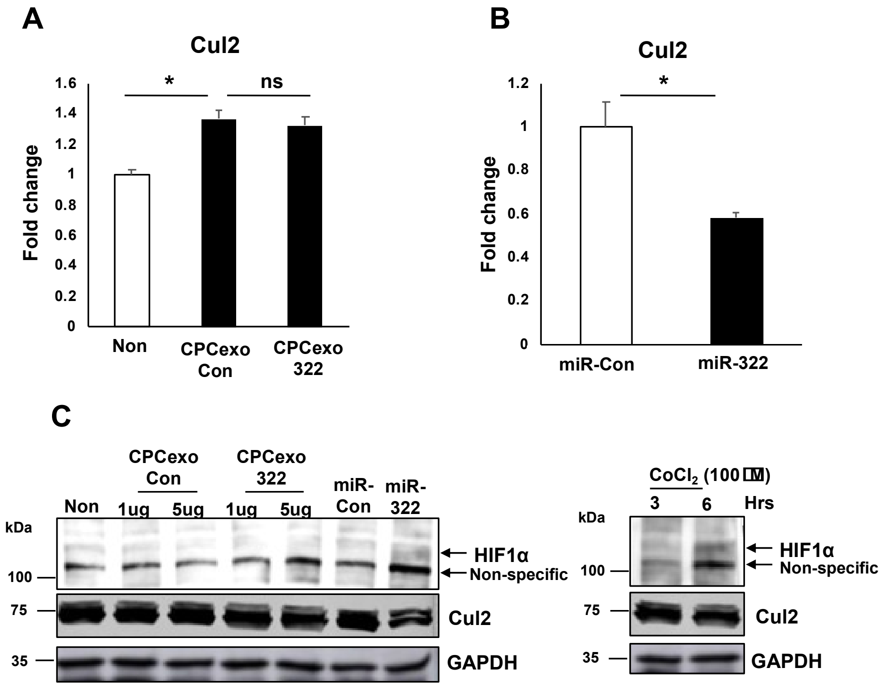

Supplementary Figure 1. Effects of CPCexo-322 and miR-322 on Culin 2 (Cul2) expression. (A-B) The mRNA level of Cul2 in CPCexo treated (**A**) and miRNA transfected (**B**) HUVECs. (**C**) Western blots of HIF1α and Cul2 expression in HUVECs treated with CPCexo-Con or CPCexo-322 or transfected with miR-Con or miR-322 (left) or treated with $CoCl_2$ (right, as a positive control for HIF1α induction in normoxic condition).

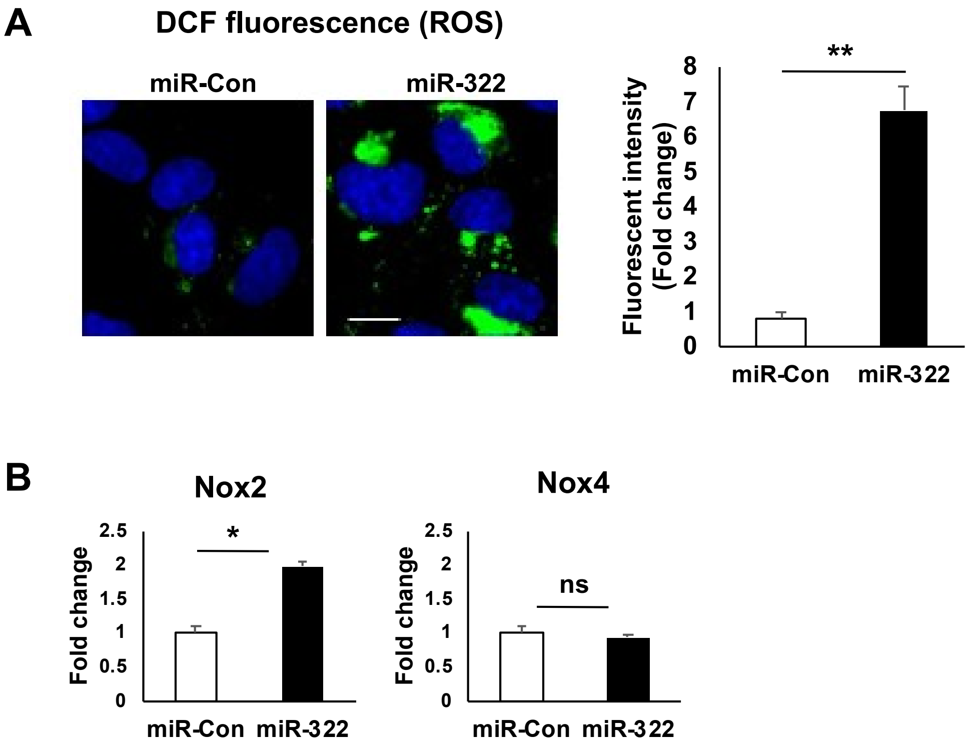

Supplementary Figure 2. miR-322 increases ROS via upregulation of Nox2 in ECs. (**A**) DCF fluorescence in HUVECs treated with miR-Con or miR-322. Nuclei were stained with DAPI (Blue). The scale bar is 10μm. Fluorescence intensity was measured using Image J. (**B**) Nox2 or Nox4 mRNA expression in HUVECs treated with either miR-Con or miR-322. Graphs represent the fold change from control (miR-Con-treated group). (n = 3, *P < 0.05, **P < 0.01 (miR-Con vs miR-322), and ns; not significant).

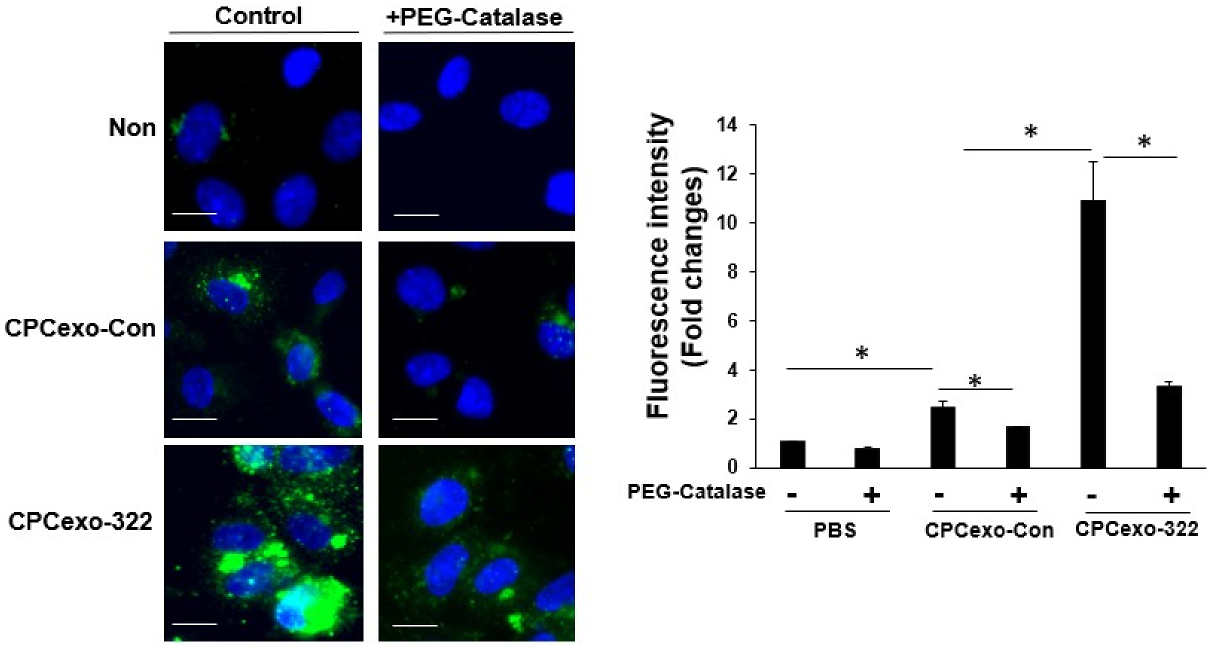

Supplementary Figure 3. Effects of PEG-catalase on H_2O_2 levels in HUVECs. HUVECs were exposed to CPCexo-Con or CPCexo-322 and increases in DCF-DA fluorescence measured. Left, Representative DCF-DA fluorescence in HUVECs. Nuclei were stained with DAPI (Blue). The scale bar is 10 μm. Right, DCF fluorescence intensity was expressed as fold change from basal (PBS only) and measured using Image J. (n = 4, *$P < 0.05$).
